# Double Plating for Complex Proximal Humeral Fractures: Clinical and Radiological Outcomes

**DOI:** 10.3390/jcm12020696

**Published:** 2023-01-16

**Authors:** Philipp A. Michel, Michael J. Raschke, J. Christoph Katthagen, Benedikt Schliemann, Isabelle Reißberg, Oliver Riesenbeck

**Affiliations:** 1 Department of Trauma-, Hand- and Reconstructive Surgery, University Hospital Muenster, 48149 Muenster, Germany; 2 Trauma-, Hand- and Orthopaedic Surgery, Herz-Jesu Krankenhaus Münster-Hiltrup, 48149 Muenster, Germany

**Keywords:** proximal humeral fracture, ORIF, double plating, dual plating

## Abstract

Double plating for proximal humeral fractures (PHF) is an option to increase the primary fixation stability. Clinical data is missing for assessment of clinical and radiological outcome, as well as complications. We retrospectively examined 35 patients with unilateral PHF, who were treated with double plating for PHF between 2013 and 2019. The mean age was 59.5 ± 12 years and the leading fracture type was a varus dislocation (Resch type IV in 55.3%). A head-split was present in 22.9% of the cases. The primary outcome measurement was the radiological neck shaft angle (NSA). The radiological follow-up was 21 ± 16.6 months and the NSA did not differ between the intraoperative and follow-up time point (131.5 ± 6.9° vs. 136.6 ± 13.7°; *p* = 0.267). The clinical follow-up was 29.5 ± 15.3 months. The Constant-score was 78.5 ± 17 points, the simple-shoulder-test (SST) was 9.3 ± 3.2 points and the subjective shoulder value (SSV) was 78.8 ± 19.5%. The over-all complication rate was 31.4%, and without stiffness 14.3%. An avascular necrosis occurred in two patients (5.7%). In conclusion, this study shows good radiological and functional outcomes after double plating of highly complex proximal humeral fractures, while the complication rate is comparable to the literature. Double plating is a viable option especially for younger patients with complex fractures as a potential alternative to fracture arthroplasty.

## 1. Introduction

The proximal humeral fracture (PHF) is one of the top three fragility fractures. The incidence in the age group >65 years is 140/100,000 for men and 356/100,000 for women and has been rising over the past years [1]. The optimal treatment is still under debate and involves non-operative treatment, open reduction and internal fixation (ORIF), hemiarthroplasty (HA) and reverse shoulder arthroplasty (RSA) [2,3]. Depending on factors such as patient age, gender, fracture type and rotator cuff status, the treatment decision can be difficult and is also influenced by surgeon preference. Additionally, a variety of outcomes and complications have been reported in the literature [4]. Regarding operative treatment, there has been a trend towards RSA for comminuted fractures in the elderly, along with a decreasing trend in HA [5,6]. The functional outcome after RSA seems to be significantly better and more reproducible compared to HA [7,8].

**Figure 1:**
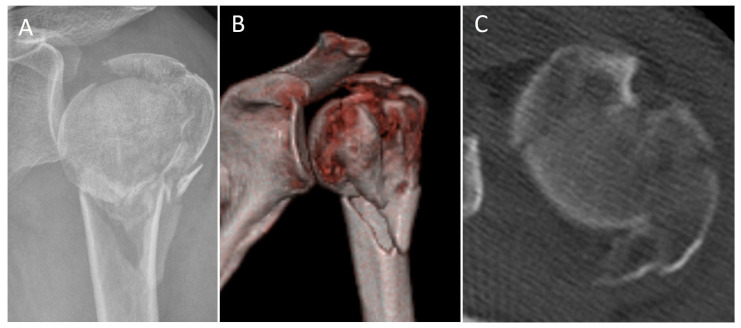
(**A**,**B**): X-ray and 3D CT-scan of a 59 year old female patient with a Resch 4GL type PHF. (**C**): Axial CT-scan shows the head-split of the articular surface type III accord to Scheibel et al. [9].

The treatment of complex PHF in middle-aged patients (50–65 years) remains particularly challenging, where RSA might not be the optimal primary treatment choice (Figure 1). ORIF with locked plating can be very demanding, especially for comminuted fractures with an additional head-split of articular surface [9,10]. It still yields a high post-operative complication and reoperation rate between 15–35% [11,12,13]. Common complications include osteonecrosis, malunion of the tuberosities, screw protrusion and varus collapse [14]. Several studies have demonstrated the importance of an anatomical fracture reduction and fixation in order to reduce complications [15,16]. Different techniques such as fibular allografts [17,18], screw tip augmentation [19,20] and double plating [21] have been described to enhance medial calcar support [22,23,24].

Double plating is a well-established technique for fractures of the upper extremities such as the distal humerus [25,26]. For the proximal humerus, it is still a relatively new concept. Biomechanical studies indicate that a locked plate in combination with an additional anteriorly placed conventional plate can enhance construct stiffness and reduce fragment movement [27,28]. Clinical data is still limited. We therefore aimed to investigate the clinical and radiographic outcomes after PHF treated with double plating. We hypothesized, that the radiological neck-shaft-angle (NSA) would not differ significantly intraoperatively and during follow-up.

## 2. Materials and Methods

This retrospective case series was conducted at a level-1 trauma center. It was approved by the responsible ethical review committee (Ärztekammer Westfalen-Lippe, 2019-158-f-S) prior to data collection. All patients with unilateral PHF, who were treated with double plating between June 2013 and June 2019, were invited for a clinical and radiographic follow-up examination.

**Figure 2:**
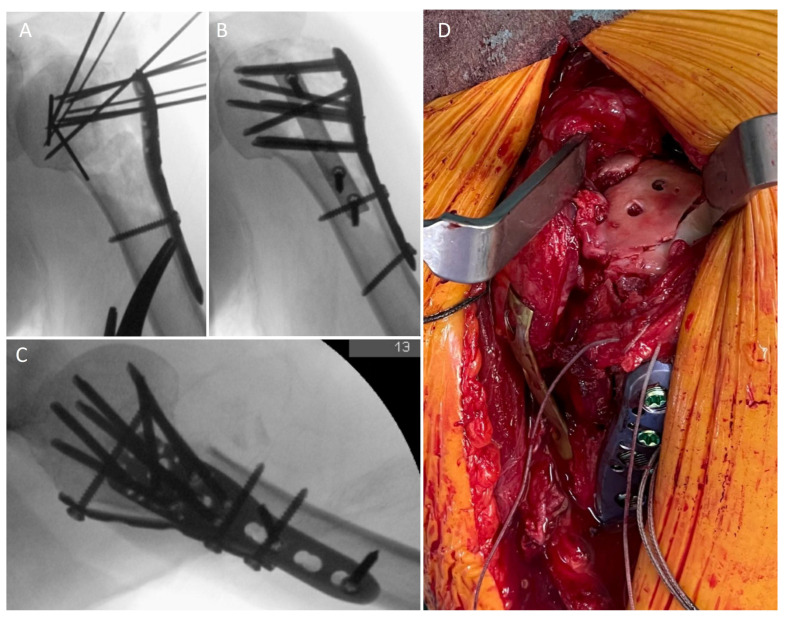
(**A**–**C**): Intraoperative X-rays demonstrating the reduction and fixation using the double plating technique of the patient in Figure 1. (**D**): Intraoperative clinical picture showing an opened-up rotator interval to control the reduction of the articular surface. The one-third tubular plate is placed on the lesser tuberosity.

The surgical technique has been described in detail [21]. Briefly, a delto-pectoral standard approach was used. Reduction was performed adapted to the fracture morphology and a locked standard plate (PHILOS, DePuy Synthes, Umkirch, Germany) was placed laterally. A one-third tubular plate (usually 5-holes) was bended according to the anatomic situation and placed anteriorly. Proximally, the plate was positioned directly onto the insertion of the subscapularis tendon at the lesser tuberosity. Distally, the plate was then fixed to the shaft with cortical screws just beneath the pectoralis major tendon to achieve a buttress effect. The long head of the biceps tendon may be cut prior to plate placement and a tenodesis to the conjoined tendons can be performed (Figure 2). The postoperative treatment included a load-restriction for 6 weeks. A sling was only used if necessary to reduce pain. Free range of motion was allowed and additionally a continuous passive motion (CPM) device was prescribed.

Inclusion criteria were all patients above the age of 18 with an unilateral PHF treated with double plating and a minimum follow-up time of 12 months. Exclusion criteria were defined as the following: previous trauma, known degenerative diseases or previous surgery at the affected shoulder; other medical conditions that limit shoulder movement (e.g., hemiparesis after stroke); pharmacological immunosuppression; lack of compliance.

The identified patients were invited for clinical and radiographic examination. Primary endpoint was the neck shaft angle (NSA). It was measured as previously described using available a.p. radiographs at the intraoperative timepoint and at the follow-up [29]. For the clinical examination, a standardized questionary was used. The range of motion was measured at the standing patient with a goniometer.

Additionally. the Constant-score, simple-shoulder-test (SST) and the subjective shoulder value (SSV) was utilized. Pain was assessed using the numerical rating scale. Clinical tests according to Neer and Hawkins were used for impingement. The Yergason test was used to assess the long head of the biceps tendon. Abduction strength was determined with the help of the device IsoForceControl EVO2 (Medical Device Solutions, Oberburg, Schweiz).

Complications were defined as the following: humeral head necrosis, infection, non-union, varus collapse, screw perforation, secondary fracture dislocation, implant failure and postoperative shoulder stiffness. In addition, the reoperation rate was recorded.

Data were analyzed with GraphPad (GraphPad Prism 8.3.0, San Diego, CA, USA). Mean, standard deviation and range were presented where applicable. The Wilcoxon signed-rank test was utilized for group comparison.

## 3. Results

### 3.1. Recruitment

We identified 66 patients that had been treated with double plating for unilateral PHF from June 2013 to June 2019, but 25 patients had to be excluded because of the following reasons: 8 patients were dead, 13 patients refused to participate, mainly because of the hospital restrictions during the COVID-19 pandemic and 4 patients met one of the above mentioned exclusion criteria (1 patient with bilateral PHF, 1 patient with intermuscular PHF, 2 patients with lack of compliance due to dementia). Of the remaining 41 patients, 6 were not contactable (lost to follow-up) and 35 (85.4%) were available for follow-up examination. Because of the active COVID-19 restrictions, 10 patients (28.6%) were assessed online with a certified software (CGM ELVI, La-Well Systems GmbH, Bünde, Germany). This included a self-deployed strength measurement [30].

### 3.2. Epidemiology

The mean clinical follow-up was 29.5 ± 15.3 months (range 12–78 months). 21 patients (60%) achieved a minimum clinical follow-up of 24 months. The mean age of the cohort was 59.5 ± 12 years (range 32–81 years), of which 16 patients (45.7%) were females and 19 patients (54.3%) males. The main cause for trauma was fall at ground level (37.1%) followed by sports (25.7%) and traffic accidents (20%) or other causes (17.1%) and 24 patients (68.6%) suffered at least from one medical pre-existing condition, most often hypertension (25.7%). In addition, 11 patients (31.4%) had active pharmacological anticoagulation. Pre-existing osteoporosis was documented for 8 patients (22.9%) and 15 patients (42.9%) had a history of prior smoking, while 6 were active smokers (17.1%).

### 3.3. Surgical Data

Fractures were graded according to the Resch classification system [31]. The leading fracture was a type IV fracture with varus dislocation in 19 patients (55.3%) (Table 1). The greater tuberosity was fractured for every patient, the lesser tuberosity for 25 patients (71.4%). A head-split was present in 8 cases (22.9%). According to the Neer classification system, every patient presented at least with a 3-part fracture.

The mean length between trauma and surgical treatment was 2.8 ± 3.6 days (range 0–14 days). For the second anterior plate, a 5-hole tubular third plate was used for 23 patients (65.7%). Other plate length included 4- (11.4%), 6- (8.6%) and 8-holes (2.9%), depending on anterior fracture extension. In one patient, a T-plate was used. Additional free screws were used for 4 patients.

Bone grafting was added for 8 patients (22.9%) including customized allogenic femoral heads (6 patients), allogenic spongiosa chips (1 patient) and an autologous iliac crest graft (1 patient).

A tenotomy and subsequent tenodesis of the long head of the biceps tendon was performed in 17 cases (48.8%). In 15 patients (42.9%) this was performed as a soft-tissue tenodesis with the conjoined tendons [32]. The mean operating time was 136 ± 45 min (range 70–238 min). The surgery was performed by specialized shoulder surgeons in 27 cases (77%).

### 3.4. Funtional Outcome

The detailed range of motion is depicted in Table 2. The operated and non-operated side differed significantly for every direction of motion (*p* < 0.001). The mean Constant-score adjusted for age and sex according to Katolik [33] was 78.5 ± 17 points for the operated side and 97.7 ± 3.5 points for the non-operated side; 19 patients achieved an excellent result (54.3%, >86 points), 7 patients a good result (20%, 71–85 points) and 5 patients a satisfactory result (14.3%, 56–70 points). In 4 patients, a poor result was documented (11.4%, <56 points) [34] and 2 of these patients had developed a humeral head necrosis and were revised with a RSA and hemi-arthroplasty.

The mean score for the simple-shoulder-test (SST) was 9.3 ± 3.2 points. The mean score for the subjective shoulder value (SSV) was 78.8 ± 19.5%. The mean NRS for pain in neutral position was 0.6 ± 1.3, while 29 patients reported no pain (80%, 0 on the NRS). The mean NRS for pain under exercise was 1.8 ± 2.3, while 21 patients reported no pain (60%, 0 on the NRS).

During the clinical assessment (*n* = 25) 9 patients (25.7%) showed signs for a capsular stiffness, 5 patients (14.3%) reported tenderness on palpation of the lesser tuberosity and 4 patients (11.4%) had positive impingement tests (subacromial).

Out of 34 patients who did sports prior to trauma, 29 (85.3%) were able to return to sports. In addition, 18 of the 35 patients were still working, 15 (83.3%) of them were able to return to their previously conducted work and 11 patients (31.4%) still received physiotherapy on a regular basis.

### 3.5. Radiological Outcome

The mean radiological follow-up was 21 ± 16.6 months (range 12–72 months). The intraoperative NSA could be obtained for 32 patients. The mean NSA was 131.5 ± 6.9° (range 121–145°). Follow-up radiological data could be gathered for 25 patients (71.4%). The mean NSA was 136.6 ± 13.7°. No significant difference could be detected between these groups (*p* = 0.267). Fracture union after one year was achieved for 20 out of 22 evaluable patients (91%).

### 3.6. Complications

A total of 14 complications were recorded for 11 out of 35 patients (31.4%). The detailed complications are shown in Table 3.

One patient with humeral head necrosis was converted to RSA 1.5 years after the initial operation. One patient with infection was treated initially with two debridements and implant retention, then 10 months postoperatively the implants were removed after fracture union. At 1.5 years after the initial operation, the patient developed a humeral head necrosis and was converted to a hemi-arthroplasty. One patient presented with a non-union after 10 months, which was treated by extra-corporal shockwave therapy. A CT scan 8 months after this procedure documented a union of the fracture. Two patients presented early with a secondary fracture dislocation. They were treated with conversion to RSA and revision ORIF 3 and 10 days after the initial surgery. Additionally one of these patients received an arthroscopic arthrolysis and implant removal after 9 months because of stiffness [35]. One patient presented with an implant failure (screw breakage) and a secondary dislocation of the fracture non-union after 7 months and was treated with revision ORIF. 6 patients had a limited range of motion postoperatively and were treated with arthroscopic arthrolysis and implant removal on average 10 ± 1.2 months after the initial operation. The revision operations are summarized in Table 4.

In summary 12 revision operations were performed for 10 patients (28.6%). Screw perforation or varus dislocation could not be detected for any patient.

## 4. Discussion

The most important finding of this study is that double plating of complex proximal humeral fractures results in stable fracture fixation with constant NSA. The complication and reoperation rates are comparable to the literature.

The reconstruction of the NSA and the enhancement of medial support in the calcar region is a key factor when treating PHF [24]. Biomechanical studies have demonstrated the effectiveness of an anterior plate, especially for four-part fractures, to increase construct stability [27,28,36,37]. The mean NSA remained stable in our cohort from intraoperatively 131.5 ± 6.9° to 136.6 ± 13.7° at follow-up. These values are comparable to other studies [38,39,40]. Schnetzke et al. found, that the quality of reduction independently influences the outcome after locked-plate fixation of complex humeral fractures and is therefore an important surgical factor for treatment success [29].

The study cohort was comparable concerning age to other studies examining ORIF for PHF. Alrabaa et al. presented a large cohort of surgically treated PHF and found a mean age of 62.0 ± 14.4 years in the ORIF group, while our cohort had a mean of 59.5 ± 12 years [5]. Concerning sex, we had an unusually low proportion of female patients. The ORIF group of the aforementioned study displayed 73.5% females, while we had 45.7% females. Taken together we tended towards a younger and male study population. Warnhoff et al. observed a similar tendency with their cohort of 25 patients with PHF treated with double plating [41]. Additionally we found a high rate of Resch type IV fractures (55.3%), type V (11.4%) and head-split fractures (22.9%). Scheibel et al. state that head-split fractures account for less than 5% of PHF [9]. Double plating is therefore a viable option in patients with a head-split PHF in order to achieve anatomic reduction and stable fixation. Especially in younger patients, ORIF yields better functional outcomes compared to hemi-arthroplasty [42,43].

**Figure 3:**
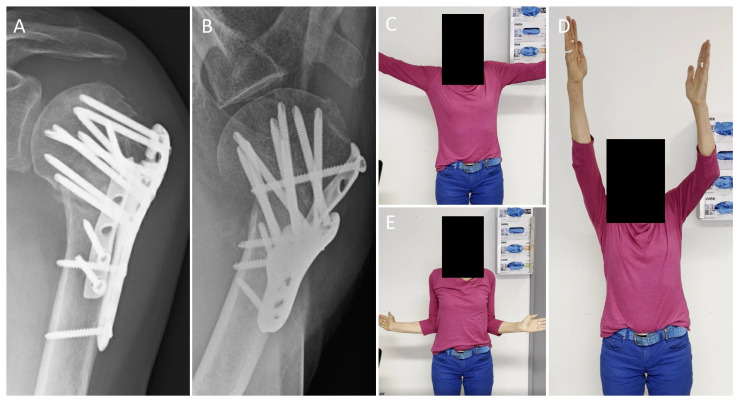
Radiological follow up after 5 months ap- (**A**) and axial (**B**) views. (**C**–**E**): Functional outcome after 5-months (same patient as Figure 1 and Figure 2).

Functional outcomes in our cohort were good. Constant scores, range of motion and pain levels are comparable to the literature. Yahuaca et al. presented a cohort of 211 patients who were treated with locked plating. The mean forward flexion at the 1 year follow-up was 130 ± 41° (in our cohort 139 ± 37°) [8] (Figure 3). Warnhoff et al. found a mean Constant score of 77 ± 17 points (in our cohort 78.5 ± 17 points) [41]. For head-split fractures, the functional outcome can be significantly worse. Peters et al. examined a cohort of 20 patients with head-split PHF treated with locked plating. The mean Constant score was 47 ± 27 points [10].

The overall complication rate of 31.4% is comparable to other cohorts examining complex PHF. Samborski et al. presented 23 patients treated with locked plating for type C (AO classification) PHF. They found a complication rate of 34.8%., three patients presented with avascular necrosis and a total of four patients hat to be converted to RSA [11]. Robinson et al. assessed a cohort of 368 patients with complex PHF (three- or four-part fractures in 77.2% of the cases) treated with ORIF with a mean age of 55.3 years, and 88 patients underwent a total of 106 repeat surgical interventions, which results in a complication rate of 23.9%. The main reason for reoperation was postoperative stiffness (17.9%). The authors state that, when reoperations for stiffness were excluded, the complication rate dropped to 8.4% [44]. When excluding stiffness for our cohort, the resulting complication rate was 14.3%. Warnhoff et al. reported a similar complication rate without stiffness of 16% [41].

An argument against the use of an anterior plate is the extended soft-tissue exposure, which could potentially worsen the humeral head vascularization and increase the rate of humeral head necrosis. Warnhoff et al. found, in their cohort of 25 patients, two (8%) with humeral head necrosis after double plating for PHF. They concluded that the necrosis rate is higher compared to the literature and ascribe this to the fracture morphology, with a destroyed medial calcar for all their cases. In fact, several studies have demonstrated the influence of the length of medial hinge on the risk for osteonecrosis [45]. In our cohort, we found two patients with avascular necrosis. This is comparable to values given in the literature, where the rate can go up to 20% for revision cases [46]. Combining the aforementioned cohorts (4/60 patients, 6.7%), it seems that double plating does not lead to an unusual high rate of avascular necrosis. Additionally, we did not examined any specific problems with the anterior plate location concerning sub-coracoidal impingement or affection of the subscapularis tendon or muscle function.

The limitations of the study include the retrospective study design and the lack of a control group. Further prospective studies with larger patient numbers are needed to verify the value of the double plating technique for PHF.

## Figures and Tables

**Table 1:** Fracture classification according to the Resch system.

Resch Type	Patients	Percentage
I	0	0%
II	1	2.9%
III	11	31.4%
IV	19	55.3%
V ^1^	4	11.4%

^1^ Anterior and posterior.

**Table 2:** Range of motion.

Movement	Operated Side	Non-Operated Side
Forward flexion	139 ± 37°	169 ± 13°
Retroversion	41 ± 16°	45 ± 13°
Abduction	128 ± 39°	168 ± 17°
Adduction	34 ± 12°	42 ± 11°
External rotation ^1^	61 ± 24°	78 ± 13°
Internal rotation ^1^	34 ± 24°	55 ± 19°

^1^ Measured in 90° Abduction.

**Table 3:** Complications.

Complication	Number
Humeral head necrosis	2 (5.7%)
Infection	1 (2.9%)
Non-union	2 (5.7%)
Secondary dislocation	2 (5.7%)
Implant failure	1 (2.9%)
Stiffness	6 (17.1%)

**Table 4:** Revision operations.

Revision Operation	Number
Debridement and implant retention	1 (2.9%)
Conversion to RSA	2 (5.7%)
Conversion to hemi-arthroplasty	1 (2.9%)
Revision ORIF	2 (5.7%)
Arthroscopic Arthrolysis + Implant removal	6 (17.1%)

## Data Availability

The data presented in this study are available on request from the corresponding author.
